# 3-Carb­oxy­quinolin-1-ium-2-carboxyl­ate monohydrate

**DOI:** 10.1107/S1600536812006988

**Published:** 2012-02-24

**Authors:** Xing Wang, Chun-Bo Liu, Yong-Sheng Yan, Shen-Tang Wang, Qing Zhang

**Affiliations:** a School of Chemistry and Chemical Engineering, Jiangsu University, Zhenjiang 212013, People’s Republic of China

## Abstract

The title compound, C_11_H_7_NO_4_·H_2_O, contains a 3-carb­oxy­quinolin-1-ium-2-carboxyl­ate (qda) zwitterion and one water mol­ecule. In the crystal, pairs of N—H⋯O hydrogen bonds link the mol­ecules into inversion dimers, and these dimers are further connected by O—H⋯O hydrogen bonds into a three-dimensional supra­molecular architecture. In addition, π–π inter­actions occur between pyridine and benzene rings from different qda ligands [centroid–centroid distance = 3.749 (1) Å] and the dihedral angles of the –CO_2_H and –CO_2_ groups to the quinoline system are 8.47 (3) and 88.16 (6)°, respectively.

## Related literature

For background on the use of quinoline carb­oxy­lic acid derivatives in metal organic frameworks, see: Dobrzyńska *et al.* (2004 ▶, 2005 ▶); Hu *et al.* (2007 ▶); Li & Liu (2010 ▶). For background on the role of noncovalent inter­molecular inter­actions, see: Wang *et al.* (2011 ▶). For related structures, see: Dobrzyńska *et al.* (2004 ▶); Dobrzyńska & Jerzykiewicz (2008 ▶); Odoko *et al.* (2001 ▶); Zurowska *et al.* (2007 ▶).
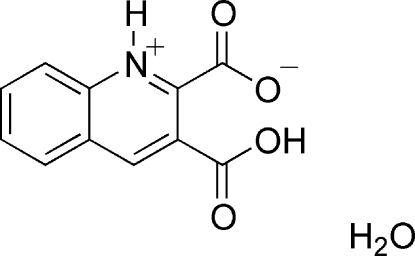


## Experimental

### 

#### Crystal data

C_11_H_7_NO_4_·H_2_O
*M* *_r_* = 235.19
Monoclinic, 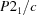

*a* = 7.5424 (15) Å
*b* = 14.422 (3) Å
*c* = 9.755 (2) Å
β = 108.17 (3)°
*V* = 1008.3 (4) Å^3^
*Z* = 4
Mo *K*α radiation
μ = 0.13 mm^−1^
*T* = 153 K
0.15 × 0.13 × 0.11 mm

#### Data collection

Rigaku CCD area-detector diffractometer
Absorption correction: multi-scan (*CrystalClear*; Rigaku, 2007 ▶) *T*_min_ = 0.981, *T*_max_ = 1
4586 measured reflections
1817 independent reflections
1547 reflections with *I* > 2σ(*I*)
*R*_int_ = 0.020

#### Refinement

*R*[*F* ^2^ > 2σ(*F* ^2^)] = 0.036
*wR*(*F* ^2^) = 0.101
*S* = 1.05
1817 reflections
168 parameters
5 restraints
H atoms treated by a mixture of independent and constrained refinement
Δρ_max_ = 0.20 e Å^−3^
Δρ_min_ = −0.20 e Å^−3^

### 

Data collection: *CrystalClear* (Rigaku, 2007 ▶); cell refinement: *CrystalClear*; data reduction: *CrystalClear*; program(s) used to solve structure: *SHELXS97* (Sheldrick, 2008 ▶); program(s) used to refine structure: *SHELXL97* (Sheldrick, 2008 ▶); molecular graphics: *CrystalClear* (Rigaku, 2007 ▶) and *DIAMOND* (Brandenburg, 1998 ▶); software used to prepare material for publication: *SHELXTL* (Sheldrick, 2008 ▶).

## Supplementary Material

Crystal structure: contains datablock(s) global, I. DOI: 10.1107/S1600536812006988/zj2053sup1.cif

Structure factors: contains datablock(s) I. DOI: 10.1107/S1600536812006988/zj2053Isup2.hkl

Supplementary material file. DOI: 10.1107/S1600536812006988/zj2053Isup3.cml

Additional supplementary materials:  crystallographic information; 3D view; checkCIF report

## Figures and Tables

**Table 1:** Hydrogen-bond geometry (Å, °)

*D*—H⋯*A*	*D*—H	H⋯*A*	*D*⋯*A*	*D*—H⋯*A*
N1—H1*A*⋯O2^i^	0.93 (2)	1.71 (2)	2.6392 (16)	170.7 (17)
O1*W*—H1*C*⋯O1^ii^	0.86 (2)	1.93 (2)	2.7589 (16)	161 (2)
O1*W*—H1*D*⋯O1	0.87 (2)	1.90 (2)	2.7597 (16)	175 (2)
O4—H4*A*⋯O1*W*^iii^	0.89 (2)	1.70 (2)	2.5950 (17)	175.6 (19)

Symmetry codes: (i) 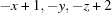; (ii) 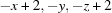; (iii) 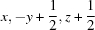.

**Fig. 1.**
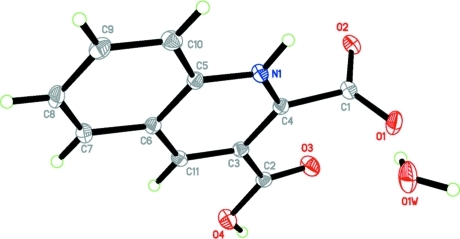
The crystal structure of the title compound with atom-labelling scheme, showing 30% probability displacement ellipsoids. H atoms are presented as a small spheres of arbitrary radius.

**Fig. 2.**
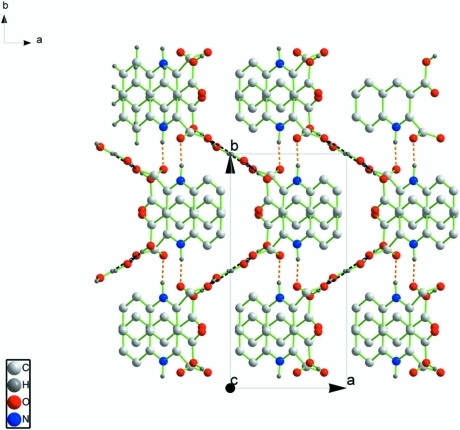
A view of 3D supramolecular architecture formed by N—H···O hydrogen bond (yellow dotted line) and O—H···O hydrogen bond (black dotted line) along c axis.

**Fig. 3.**
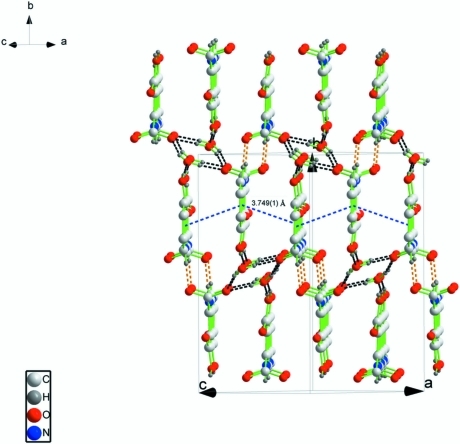
A view of the formation of the O—H···O interactions and π–π interactions.

## supplementary crystallographic information

### Comment

Quinoline carboxylic acid derivatives have been explored in the synthesis of metal organic frameworks due to their abundant coordination modes which lead to the construction of metal organic frameworks with intriguing structures and functional properties (Dobrzyńska *et al.* 2004; Hu *et al.* 2007; Dobrzyńska *et al.* 2005; Li & Liu 2010). It is well known that noncovalent intermolecular interactions such as hydrogen bonding interactions and π–π interactions, play crucial role in the design and construction of supramolecular architecture (Wang *et al.* 2011). Taking quinoline-2-carboxylic acid for example, the crystal structures of its metal complexes have been determined for several metal ions, including Cu^II^ (Zurowska *et al.* 2007), Mn^II^ (Dobrzyńska & Jerzykiewicz, 2008), Ni^II^ (Odoko *et al.* 2001), Co^II^ and Fe^II^ (Dobrzyńska *et al.* 2004). Of these complexes, the magnetic properties of Cu^II^, Co^II^ and Fe^II^ complexes have also been investigated. Herein, the structurally similar quinoline-2,3-dicarboxylic acid (qda) is a good choice for constructing a framework with novel physical properties, and the crystal structure of its monohydrate is reported now.

In this report, the title compound was prepared by using quinoline-2,3-dicarboxylic acid (qda) ligand under hydrothermal conditions. The analysis of crystal structure shows that one proton of carboxyl group of qda is transferred to N atom from pyridine ring, and one water molecule exists in the crystal lattice (Fig. 1). As shown in Fig. 2, the N—H···O hydrogen bond (yellow dotted line) links the molecules into dimers, and these dimers are further connected by O–H···O hydrogen bond (black dotted line) to a 3D supramolecular architecture. In addition, the π–π interactions (blue dotted line) occur between pyridine ring and benzene ring from different qda ligands with the distance of 3.749 (1) Å, making the supramolecular network more stable (Fig. 3).

### Experimental

The quinoline-2,3-dicarboxylic acid (qda) was purchased commercially and used without further purification. A mixture of ZnCl_2_ (13.4 mg, 0.1 mmol), and qda (43.8 mg, 0.2 mmol) was dissolved in a 15 mL of water, and the pH was adjusted to 7 by 1 mol L^-1^ sodium hydroxide solution. Then the mixture was placed in a 25 mL autoclave with Teflon-liner. The autoclave was heated to 433 K and held at this temperature for three days. It was then cooled to room temperature under spontaneous conditions. The colourless block crystals were obtained with a yield of 60 %, however, X-ray crystallographic study shows that this crystal is not zinc complex but the title compound.

### Refinement

All H atoms on C atoms were placed in an ideal position using a riding model th with C—H distances of 0.93 Å and *U*_iso_(H)=1.2*U*_eq_(C). The pyridinium NH and hydroxy H-atoms were located in a difference Fourier map with N—H distance of 0.933 Å and O—H distance of 0.893 Å, and their temperature factors were freely refined. Water hydrogen atoms were also located in a difference Fourier map with a distance restraint to their parent O atoms (0.867 and 0.858 Å, respectively) and *U*_iso_(H)=1.5*U*_eq_(O).

### Crystal data

**Table:**

C_11_H_7_NO_4_·H_2_O	*F*(000) = 488
*M**_r_* = 235.19	*D*_x_ = 1.549 Mg m^−^^3^
Monoclinic, *P*2_1_/*c*	Mo *K*α radiation, λ = 0.71073 Å
Hall symbol: -P 2ybc	Cell parameters from 4018 reflections
*a* = 7.5424 (15) Å	θ = 4.0–28.9°
*b* = 14.422 (3) Å	µ = 0.13 mm^−^^1^
*c* = 9.755 (2) Å	*T* = 153 K
β = 108.17 (3)°	Prism, colourless
*V* = 1008.3 (4) Å^3^	0.15 × 0.13 × 0.11 mm
*Z* = 4	

### Data collection

**Table:**

Rigaku CCD area-detector diffractometer	1817 independent reflections
Radiation source: fine-focus sealed tube	1547 reflections with *I* > 2σ(*I*)
Graphite monochromator	*R*_int_ = 0.020
Detector resolution: 28.5714 pixels mm^-1^	θ_max_ = 25.3°, θ_min_ = 4.0°
ω scans	*h* = −8→7
Absorption correction: multi-scan (*CrystalClear*; Rigaku, 2007)	*k* = −17→15
*T*_min_ = 0.981, *T*_max_ = 1	*l* = −8→11
4586 measured reflections	

### Refinement

**Table:**

Refinement on *F*^2^	Primary atom site location: structure-invariant direct methods
Least-squares matrix: full	Secondary atom site location: difference Fourier map
*R*[*F*^2^ > 2σ(*F*^2^)] = 0.036	Hydrogen site location: inferred from neighbouring sites
*wR*(*F*^2^) = 0.101	H atoms treated by a mixture of independent and constrained refinement
*S* = 1.05	*w* = 1/[σ^2^(*F*_o_^2^) + (0.0638*P*)^2^ + 0.0617*P*] where *P* = (*F*_o_^2^ + 2*F*_c_^2^)/3
1817 reflections	(Δ/σ)_max_ = 0.001
168 parameters	Δρ_max_ = 0.20 e Å^−^^3^
5 restraints	Δρ_min_ = −0.20 e Å^−^^3^

### Special details

**Table:**

Geometry. All esds (except the esd in the dihedral angle between two l.s. planes) are estimated using the full covariance matrix. The cell esds are taken into account individually in the estimation of esds in distances, angles and torsion angles; correlations between esds in cell parameters are only used when they are defined by crystal symmetry. An approximate (isotropic) treatment of cell esds is used for estimating esds involving l.s. planes.
Refinement. Refinement of F^2^ against ALL reflections. The weighted R-factor wR and goodness of fit S are based on F^2^, conventional R-factors R are based on F, with F set to zero for negative F^2^. The threshold expression of F^2^ > 2sigma(F^2^) is used only for calculating R-factors(gt) etc. and is not relevant to the choice of reflections for refinement. R-factors based on F^2^ are statistically about twice as large as those based on F, and R- factors based on ALL data will be even larger.

### Fractional atomic coordinates and isotropic or equivalent isotropic displacement parameters (Å^2^)

**Table:**

	*x*	*y*	*z*	*U*_iso_*/*U*_eq_	
C1	0.65319 (19)	0.09213 (9)	1.05483 (14)	0.0216 (3)	
C2	0.67451 (18)	0.30200 (9)	1.06906 (15)	0.0216 (3)	
C3	0.54746 (17)	0.25508 (9)	0.93895 (14)	0.0195 (3)	
C4	0.54008 (17)	0.15740 (9)	0.93584 (14)	0.0194 (3)	
C5	0.31108 (17)	0.16028 (10)	0.70347 (14)	0.0199 (3)	
C6	0.31153 (18)	0.25796 (9)	0.70374 (14)	0.0192 (3)	
C7	0.19098 (18)	0.30475 (10)	0.58263 (14)	0.0231 (3)	
H7	0.1883	0.3692	0.5804	0.028*	
C8	0.07928 (19)	0.25556 (10)	0.46975 (16)	0.0255 (3)	
H8	0.0000	0.2867	0.3909	0.031*	
C9	0.08227 (19)	0.15775 (10)	0.47086 (15)	0.0266 (3)	
H9	0.0054	0.1253	0.3923	0.032*	
C10	0.19651 (19)	0.10985 (10)	0.58570 (15)	0.0254 (3)	
H10	0.1983	0.0454	0.5858	0.031*	
C11	0.43333 (17)	0.30389 (9)	0.82448 (14)	0.0197 (3)	
H11	0.4362	0.3684	0.8265	0.024*	
O1	0.80640 (13)	0.06645 (7)	1.04539 (11)	0.0322 (3)	
O2	0.57317 (13)	0.06656 (6)	1.14257 (10)	0.0241 (3)	
O3	0.75594 (15)	0.25971 (7)	1.17666 (11)	0.0346 (3)	
N1	0.42599 (15)	0.11485 (8)	0.82176 (11)	0.0206 (3)	
H1A	0.418 (2)	0.0503 (15)	0.8245 (19)	0.046 (5)*	
O1W	0.88294 (16)	0.02969 (8)	0.79197 (12)	0.0380 (3)	
H1C	0.987 (2)	0.0010 (13)	0.825 (2)	0.057*	
H1D	0.853 (3)	0.0434 (14)	0.8685 (19)	0.057*	
O4	0.68389 (15)	0.39253 (7)	1.05383 (12)	0.0300 (3)	
H4A	0.757 (3)	0.4174 (13)	1.136 (3)	0.059 (6)*	

### Atomic displacement parameters (Å^2^)

**Table:**

	*U*^11^	*U*^22^	*U*^33^	*U*^12^	*U*^13^	*U*^23^
C1	0.0257 (7)	0.0190 (7)	0.0174 (7)	0.0003 (5)	0.0030 (6)	−0.0014 (5)
C2	0.0206 (7)	0.0246 (7)	0.0197 (8)	−0.0019 (6)	0.0061 (6)	0.0011 (6)
C3	0.0197 (7)	0.0212 (7)	0.0184 (7)	−0.0010 (5)	0.0070 (6)	0.0002 (5)
C4	0.0184 (7)	0.0224 (7)	0.0181 (7)	0.0003 (5)	0.0067 (5)	0.0004 (5)
C5	0.0193 (7)	0.0224 (7)	0.0180 (7)	0.0006 (5)	0.0060 (5)	0.0014 (5)
C6	0.0185 (7)	0.0214 (7)	0.0183 (7)	0.0006 (5)	0.0067 (6)	0.0002 (5)
C7	0.0234 (7)	0.0226 (7)	0.0225 (8)	0.0031 (6)	0.0059 (6)	0.0028 (6)
C8	0.0210 (7)	0.0319 (8)	0.0197 (7)	0.0040 (6)	0.0006 (6)	0.0032 (6)
C9	0.0231 (7)	0.0326 (8)	0.0209 (7)	−0.0040 (6)	0.0022 (6)	−0.0049 (6)
C10	0.0280 (8)	0.0223 (7)	0.0251 (8)	−0.0034 (6)	0.0068 (6)	−0.0021 (6)
C11	0.0224 (7)	0.0183 (7)	0.0199 (8)	0.0001 (5)	0.0087 (6)	0.0007 (5)
O1	0.0256 (5)	0.0411 (6)	0.0285 (6)	0.0120 (5)	0.0063 (4)	0.0085 (5)
O2	0.0345 (6)	0.0186 (5)	0.0198 (5)	0.0016 (4)	0.0092 (4)	0.0012 (4)
O3	0.0391 (6)	0.0319 (6)	0.0220 (6)	−0.0069 (5)	−0.0061 (5)	0.0050 (5)
N1	0.0240 (6)	0.0173 (6)	0.0193 (6)	0.0012 (5)	0.0050 (5)	0.0010 (4)
O1W	0.0426 (7)	0.0441 (7)	0.0249 (6)	0.0210 (5)	0.0073 (5)	0.0095 (5)
O4	0.0367 (6)	0.0217 (5)	0.0243 (6)	−0.0046 (4)	−0.0011 (5)	−0.0029 (4)

### Geometric parameters (Å, º)

**Table:**

C1—O1	1.2439 (17)	C6—C7	1.4170 (18)
C1—O2	1.2470 (17)	C7—C8	1.359 (2)
C1—C4	1.5310 (18)	C7—H7	0.9300
C2—O3	1.2038 (16)	C8—C9	1.411 (2)
C2—O4	1.3184 (17)	C8—H8	0.9300
C2—C3	1.4929 (18)	C9—C10	1.369 (2)
C3—C11	1.3727 (18)	C9—H9	0.9300
C3—C4	1.4098 (19)	C10—H10	0.9300
C4—N1	1.3260 (17)	C11—H11	0.9300
C5—N1	1.3741 (17)	N1—H1A	0.93 (2)
C5—C10	1.4059 (19)	O1W—H1C	0.858 (15)
C5—C6	1.409 (2)	O1W—H1D	0.868 (15)
C6—C11	1.4126 (19)	O4—H4A	0.89 (2)
			
O1—C1—O2	128.49 (13)	C8—C7—H7	120.0
O1—C1—C4	115.97 (12)	C6—C7—H7	120.0
O2—C1—C4	115.32 (11)	C7—C8—C9	120.75 (13)
O3—C2—O4	124.70 (13)	C7—C8—H8	119.6
O3—C2—C3	121.96 (12)	C9—C8—H8	119.6
O4—C2—C3	113.32 (12)	C10—C9—C8	121.03 (13)
C11—C3—C4	118.97 (12)	C10—C9—H9	119.5
C11—C3—C2	122.18 (12)	C8—C9—H9	119.5
C4—C3—C2	118.81 (11)	C9—C10—C5	118.53 (13)
N1—C4—C3	119.43 (11)	C9—C10—H10	120.7
N1—C4—C1	114.47 (11)	C5—C10—H10	120.7
C3—C4—C1	126.09 (11)	C3—C11—C6	121.18 (13)
N1—C5—C10	120.37 (13)	C3—C11—H11	119.4
N1—C5—C6	118.34 (12)	C6—C11—H11	119.4
C10—C5—C6	121.29 (12)	C4—N1—C5	123.95 (12)
C5—C6—C11	118.10 (12)	C4—N1—H1A	118.0 (11)
C5—C6—C7	118.30 (12)	C5—N1—H1A	118.0 (11)
C11—C6—C7	123.60 (13)	H1C—O1W—H1D	104.2 (18)
C8—C7—C6	120.10 (13)	C2—O4—H4A	109.7 (13)
			
O3—C2—C3—C11	170.11 (13)	C5—C6—C7—C8	−0.28 (18)
O4—C2—C3—C11	−8.67 (17)	C11—C6—C7—C8	179.25 (13)
O3—C2—C3—C4	−7.72 (19)	C6—C7—C8—C9	−0.4 (2)
O4—C2—C3—C4	173.51 (12)	C7—C8—C9—C10	0.4 (2)
C11—C3—C4—N1	1.20 (18)	C8—C9—C10—C5	0.2 (2)
C2—C3—C4—N1	179.10 (11)	N1—C5—C10—C9	179.13 (12)
C11—C3—C4—C1	−178.47 (11)	C6—C5—C10—C9	−0.88 (19)
C2—C3—C4—C1	−0.57 (18)	C4—C3—C11—C6	−0.89 (18)
O1—C1—C4—N1	89.54 (14)	C2—C3—C11—C6	−178.72 (12)
O2—C1—C4—N1	−85.51 (14)	C5—C6—C11—C3	−0.38 (18)
O1—C1—C4—C3	−90.78 (16)	C7—C6—C11—C3	−179.91 (12)
O2—C1—C4—C3	94.17 (15)	C3—C4—N1—C5	−0.20 (18)
N1—C5—C6—C11	1.36 (18)	C1—C4—N1—C5	179.51 (11)
C10—C5—C6—C11	−178.63 (11)	C10—C5—N1—C4	178.89 (12)
N1—C5—C6—C7	−179.09 (11)	C6—C5—N1—C4	−1.10 (18)
C10—C5—C6—C7	0.92 (18)		

### Hydrogen-bond geometry (Å, º)

**Table:**

*D*—H···*A*	*D*—H	H···*A*	*D*···*A*	*D*—H···*A*
N1—H1*A*···O2^i^	0.93 (2)	1.71 (2)	2.6392 (16)	170.7 (17)
O1*W*—H1*C*···O1^ii^	0.86 (2)	1.93 (2)	2.7589 (16)	161 (2)
O4—H4*A*···O1*W*^iii^	0.89 (2)	1.70 (2)	2.5950 (17)	175.6 (19)
O1*W*—H1*D*···O1	0.87 (2)	1.90 (2)	2.7597 (16)	175 (2)

Symmetry codes: (i) −*x*+1, −*y*, −*z*+2; (ii) −*x*+2, −*y*, −*z*+2; (iii) *x*, −*y*+1/2, *z*+1/2.
